# Choroidal Vascular Impairment in Intermediate Age-Related Macular Degeneration

**DOI:** 10.3390/diagnostics12051290

**Published:** 2022-05-22

**Authors:** Rita Flores, Ângela Carneiro, Guilherme Neri, Ana C. Fradinho, Bruno Quenderra, Maria João Barata, Sandra Tenreiro, Miguel C. Seabra

**Affiliations:** 1 Department of Ophthalmology, Centro Hospitalar de Lisboa Central EPE, 1169-050 Lisbon, Portugal; jose.pires@chlc.min-saude.pt (G.N.); bruno.quendera@chlc.min-saude.pt (B.Q.); maria.barata2@chlc.min-saude.pt (M.J.B.); 2 NOVA Medical School, Universidade Nova de Lisboa, 1169-056 Lisbon, Portugal; ana.fradinho@nms.unl.pt (A.C.F.); stenreiro@nms.unl.pt (S.T.); miguel.seabra@nms.unl.pt (M.C.S.); 3 Department of Ophthalmology, Centro Hospitalar Universitário de São João, 4099-002 Porto, Portugal; acarneir@med.up.pt; 4 Faculty of Medicine, University of Porto, 4099-002 Porto, Portugal; 5 UCL Institute of Ophthalmology, London EC1V 9EL, UK

**Keywords:** intermediate AMD, chorioretinal vasculature, optical coherence tomography, OCT-angiography

## Abstract

Age-related macular degeneration (AMD) is a multifactorial disease, whose complete pathogenesis is still unclear. Local hemodynamics may play a crucial role in its manifestation and progression. To evaluate choroidal and retinal vascular parameters, a total of 134 eyes were analyzed, 100 with intermediate AMD and 34 age matched healthy controls. 131 eyes of 104 patients were eligible for complete image assessment and 3 eyes were excluded for insufficient image quality: Group 1: intermediate AMD (*n* = 97) and Group 2: healthy controls (*n* = 34). Spectral domain optic coherence tomography (SD-OCT) with enhanced depth imaging (EDI) and optic coherence tomography angiography (OCT-A) were acquired using Spectralis (Heidelberg Engineering). Choroid and retinal capillary plexus were evaluated and image binarization was used to obtain quantitative data. Mean age was 77.67 years old (YO) and 67.2% were women. Total subfoveal choroidal area and luminal area were significantly reduced in Group 1 compared with Group 2 (0.88 mm^2^ and 0.40 mm^2^ vs. 1.24 mm^2^ and 0.55 mm^2^, respectively) (*p* < 0.05). Regarding choriocapillary flow density, AMD eyes recorded reduced values (34.83%) compared with controls (36.25%) (*p* < 0.05). Chorioretinal vasculature is impaired in intermediate AMD patients and vascular parameters could be attractive new prognostic biomarkers. Future therapeutic approaches may target this vascular dysfunction and delay disease progression.

## 1. Introduction

Age-Related Macular Degeneration (AMD) is a chronic and progressive disorder of the macula, being the leading cause of irreversible visual impairment among older adults, affecting 30–50 million individuals worldwide [1,2,3]. AMD is defined by a progressive degeneration of the photoreceptors, retinal pigment epithelium (RPE) and choriocapillaris, leading to loss of central vision [1]. Although its pathogenesis is still unclear, there is a proven genetic background, with polymorphisms within genes encoding complement factor H, human high-temperature requirement A-1 (HTRA1), age-related maculopathy susceptibility 2 (ARMS2), complement factor B (CFB), complement C2 and C3, identified as potential predictors for AMD development [4,5]. Environmentally, the most important risk factor is smoking, and advanced age is the strongest risk factor [1].

The earliest histopathological manifestation of the disease are lipid deposits within Bruch’s membrane (BrM), possibly from reduced effectiveness of the RPE to digest the cellular debris associated with photoreceptor outer segments turnover [6]. These deposits are known as basal linear or laminar deposits and, as they evolve, thickening of the BrM collagenous layers with degeneration of elastin and collagen will predispose drusen formation, the first visible clinical sign of AMD [7]. Typical drusen appear as focal, whitish yellow excrescences, located between the RPE and BrM, varying in number, size, shape, and distribution [8].

Hard drusen are present in 80% of the general population over 30 years of age and appear as round, discrete yellow-white spots, measuring less than 63 µm, currently considered as a physiological sign of ageing [5]. Soft drusen are poorly defined, with non-discrete borders measuring more than 63 µm, being large, soft and confluent drusen associated with a higher risk for development of advanced AMD [5]. They are also associated with aging, affecting 26% of individuals over the age of 70. Cuticular drusen are yellow deposits located below the basal membrane of RPE, triangular shape, small diameter (50/75 µm) and usually distributed in clusters. Reticular pseudodrusen or subretinal drusenoid deposits (SDD) are yellow faint and interlacing network in the apical region of the RPE and their presence entails a worst prognostic, being associated with a more aggressive disease [9,10,11,12].

Previous bibliography and published articles refer AMD as a difficult issue with respect to its etiology and pathogenic mechanisms. AMD was first described in the late 19th century, and since then several hypotheses have been proposed: an inflammatory, a degenerative disease or a result of choroidal hemodynamic disturbances and ischemia. Ischemic processes have been frequently suggested to have a central role in its development and progression [13].

Progressive degeneration of macular photoreceptors is not the unique event in AMD with RPE being an important target in this pathogenic process. RPE apical extensions are in close proximity to the photoreceptors outer segments and the basal cellular pole next to BrM, separating the outer retina from the choroid. Although cellular and molecular theories of AMD have been targets of particular interest in the last decades, emerging data suggests that local hemodynamic factors may have a significant role in AMD pathologies. In fact, RPE and photoreceptors are metabolically related cells, and such close relationship is necessary to achieve a healthy status, which in turn depends primarily on choroidal blood flow as a source of oxygen and nutrients.

Changes in choroidal blood flow have been proposed as a pathogenic trigger for AMD, in the past. Possek hypothesized that atherosclerotic changes in ocular blood vessels could be a factor subjacent to neovascular AMD [14,15]. Friedman and other authors developed this hemodynamic theory further, proposing that BrM and sclera stiffening due to accumulations of lipoproteins, increases choroidal vascular resistance that subsequently promotes an increase in hydrostatic pressure or a decrease in choroidal perfusion [16,17,18,19]. Such mechanisms have some resemblance to atherosclerosis due to lipid deposition in arterial walls.

In the healthy outer retina, 85–90% of oxygen is delivered via the choriocapillaris to the outer layers, while the remaining 10–15% is obtained from the deep retinal capillary plexus [20,21]. Consequently and concerning vascular etiology for AMD, the primordial pathological changes are likely to occur in the choroidal vasculature retinal [20]. Macular area is consistently exposed to light with potential toxicity and consequently overload of oxidative stress; these factors explain the small tolerance for any homeostatic disturbance, like BrM thickening or drusen deposition [20,21]. The primary insult could occur at the level of the RPE, which leads to secondary choriocapillaris degeneration, and drusen formation could be affected by choriocapillaris dysfunction. Whether those microvascular events are a cause or a consequence of drusen remains to be determined.

Optic coherence tomography (OCT) with enhanced-depth imaging (EDI) improvement and OCT angiography (OCT-A) are noninvasive techniques that provide information concerning choroid [22] and retinal [20] circulation. Quantitative data can be obtained directly in some OCT and OCT-A devices or indirectly, using image processing tools as binarization [22,23] method, that allows relative quantification of choroid and retina vascular component. Nevertheless, there is still some misunderstanding about the best parameter to study choroidal vascular disease but relationship between vascular and stroma components (choroidal vascularity index) seems to be a promising tool [22,23,24]. Impact of subretinal drusenoid deposits in total choroidal thickness and in choroidal vascularity index has been studied in nonexudative AMD patients [24] but the authors did not specifically evaluate these factors in intermediate stages.

In this study, we describe and analyse vascular changes of the choroid and retina in intermediate AMD patients that could become new prognostic biomarkers.

## 2. Materials and Methods

### 2.1. Study Design, Study Population and Ethics Issues

Cross sectional, prospective and noninterventional study conducted in outpatient Medical Retina Clinic of the Ophthalmology department of the Central Lisbon University Hospital Center (Centro Hospitalar Universitário de Lisboa Central—CHULC), a tertiary hospital in Lisbon, Portugal. The study complied with the tenets of the Declaration of Helsinki and was approved by Institutional review board and ethics committee. All participants were asked to sign an informed consent. Inclusion criteria consider intermediate AMD patients, based on Beckman classification [1,20,25], recruited during the period of January 2019 and December 2021. This clinical classification system is based on fundus lesions assessed within 2 disc diameters of the fovea in persons older than 55 years. Persons with large drusen (≥125 μm) or with pigmentary abnormalities associated with at least medium drusen (≥63 and <125 μm) were considered to have intermediate AMD. If two eyes of the same patient were eligible we included both in the study. Patient underwent a complete ophthalmologic evaluation, including best corrected visual acuity (BCVA), intraocular pressure (IOP), slit lamp biomicroscopy and 90D indirect fundoscopy. Color fundus photography (CFP), fundus autofluorescence (FAF), near infrared reflectance (NIR), optic coherence tomography (OCT) with enhanced-depth imaging (EDI) improvement and OCT angiography (OCT-A) were performed in all patients. The presence of SDD was determined by identification of characteristic dot/ribbon shaped lesions on NIR and FAF, with further confirmation on OCT.

The control group consisted of age matched healthy subjects recruited from the refraction department at the same hospital, and without any sign of AMD (on CFP, FAF and OCT scans). The exclusion criteria included patients with less than 55 years old, with refractive error above 6 diopters (positive or negative spherical equivalent), with previous documented or known retinal disease, previous retinal surgery or laser, history of glaucoma or optic neuropathy and history of systemic illness or medication with potential retinal toxicity. Any significant media opacities and inappropriate fundus imaging were also excluded. Previous cataract surgery was not considered an exclusion criterion.

### 2.2. Image Analysis and Measurements

**Figure 1:**
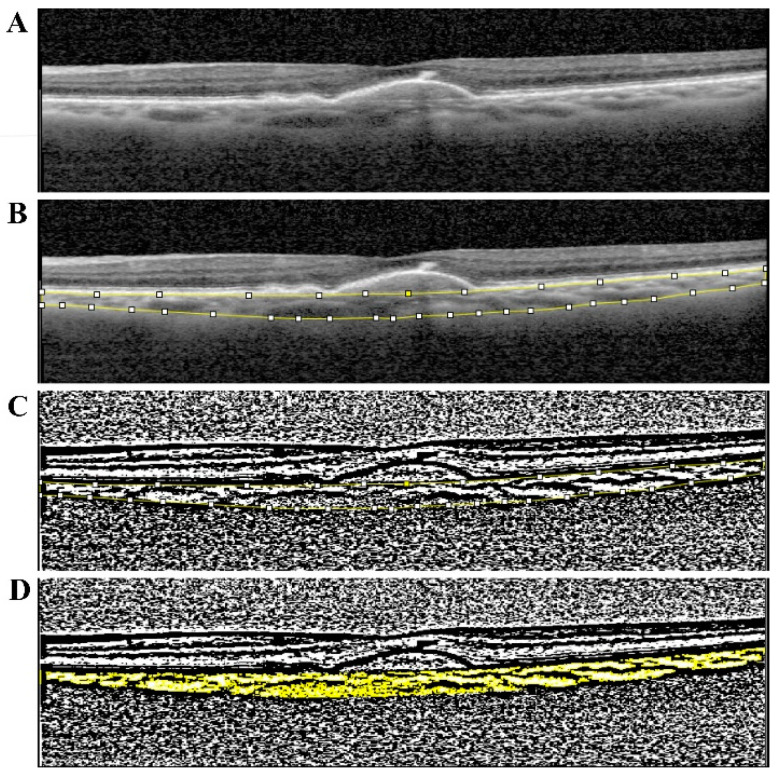
Examples of image binarization of the Choroid. (**A**) Macular SD-OCT-EDI centered on fovea and 6000 μm centered section in the fovea. (**B**) Manual measurement of the choroidal thickness. Yellow lines delimit inner boarder—Bruch’s membrane and outer boarder—escleral transition. (**C**) Image binarization—converting grey scale in binarized images. (**D**) Studied area and image software: Black pixels images (vascular lumen) and white pixels images (stroma).

Macular spectral domain OCT (SD-OCT) with enhanced depth imaging (EDI) and OCT-A, were acquired using OCT SPECTRALIS (Heidelberg Engineering, Heidelberg, Germany). The choroid was imaged using the SD-OCT-EDI mode [26]. The macular region was scanned with a wavelength of 870 nm, acquisition speed of 40.000 A-scans per second, 20° × 20° centered on the fovea, dense program, 512 × 49 scans. Each scan was spaced 120 μm apart from each other. Subfoveal choroidal thickness was measured manually using the calliper tool available in the software, using the 1:1 µm viewing mode, in the horizontal EDI scan passing through the center of the fovea (Figure 1). Choroidal thickness was defined as the vertical distance from the hyperreflective line of the Bruch’s membrane (BrM) (lower boundary of Retinal Pigment Epithelium (RPE) to the chorioscleral interface. If the hyperreflective line of the BrM was not separated from the RPE, the choroid was measured from beneath the outermost hyperreflective line of RPE/BrM layer to the chorioscleral interface [27]. Total choroidal area (TCA), luminal area (LA), stromal area (SA) and choroidal vascularity index (CVI = LA/TCA) of the subfoveal 6000 µm, were obtained after choroidal binarization performed resorting to Image J software using the scan passing through the fovea. Only this scan was selected as the region of interest, as we suppose it is a representative segment of the macular region due to the segmental nature of choroidal blood supply. Binarization imaging converts grey scale in black and white pixels images. Black pixels images in the choroid correspond to vascular lumen and white pixels images to stroma.

**Figure 2:**
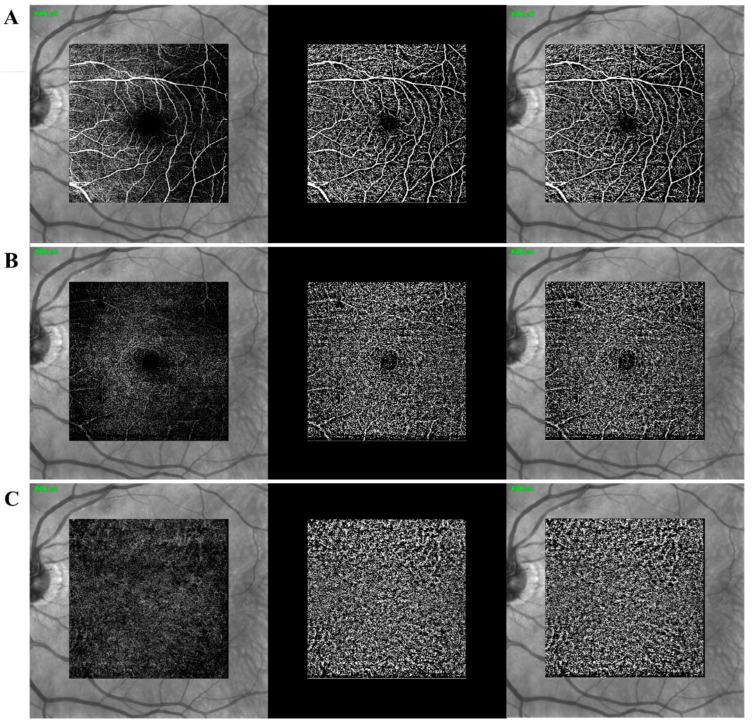
Examples of image binarization of Retinal Capillary Plexus (SCP, DCP) and Choriocapillaris (CC). (**A**) First row images: Near-infrared reflectance (NIR) and overlap composition with “enface” OCT-A images of SCP; SCP image binarization; Near-infrared reflectance (NIR) and overlap composition with SCP image binarization. (**B**) Second row images: Near-infrared reflectance (NIR) and overlap composition with “enface” OCT-A images of DCP; DCP image binarization; Near-infrared reflectance (NIR) and overlap composition with DCP image binarization. (**C**) Third row images: Near-infrared reflectance (NIR) and overlap composition with “enface” OCT-A images of Choriocapillaris; CC image binarization; Near-infrared reflectance (NIR) and overlap composition with CC image binarization.

Additionally, the OCT-A was performed with a wavelength of 870 nm, acquisition speed 40.000 A-scans per second, 20° × 20° centered on the fovea, 512 × 512 scans, spacing 11 μm. The scan with the highest quality was selected and further analysed using the same binarization software. Those binarized “enface” images of superficial capillary plexus (SCP), deep capillary plexus (DCP) and choriocapillaris area (CC) allowed to calculate vascular area (white pixels images) for each particular layer. OCT-A en face projections were defined for SCP as the projection from automatic segmented internal limiting membrane (ILM) to inner plexiform layer (IPL); DCP as the projection from automatic segmented IPL to outer plexiform layer (OPL) and CC as the projection from automatic segmented BrM with a thickness of 20 μm centered 10 μm below BrM automatic segmentation line. In those images, total measured area is constant (studied area corresponding to 20°× 20° centered on the fovea), and flow density (FD) of each particular layer represents the ratio between vessel area and total measured area (Figure 2). Imaging artefacts were assessed by two experimented ophthalmologists in this area. Motion artefacts were considered present when a white-line was detectable on the OCT-A image with concomitant lateral displacement on B-scan images. Projection artefacts as well as motion artefacts, displacement artefacts (discontinuous blood vessels), shadowing (attenuation of the signal), vessel doubling (two copies of a blood vessel), and white line artefacts were assessed on sequential OCT-A images. After each examination, the images were checked, and those with clearly visible retinal vessels and no artefacts were selected.

### 2.3. Statistical Analyses

Normality of the test was analyzed in Q-Q box and with Shapiro Wilk test, and the sample showed a normal distribution in most but not in all the variables. The group comparison was performed with known parametric test t-student for most of the studied variables and with nonparametric Mann-Whitney U test (Wilcoxon rank-sum test for clustered data) and Kruskal-Wallis test for TCA, LA and CVI, which showed non normal distribution. SPSS software (Version 26, Statistical Package for Social Science IBM) was used for statistics analysis and to generate box-and whisker plots; values of *p* < 0.05 were considered statistically significant.

Further data analysis was performed in Python using the application Jupyter Notebooks version 6.4.5 from Anaconda^®^. Linear regression equations and respective coefficients of determination (R-squared) were calculated to assess the linear relationship and the strength of the model, respectively, between total choroidal and luminal areas of healthy and AMD patients. Additionally, Spearman’s rank correlation coefficient (ρ) was calculated between choriocapillary flow density and the luminal choroidal area for patients and controls.

This statistical measure indicated the strength of a monotonic relationship between the paired data and statistical significance (*p*-value) was calculated for each correlation coefficient.

## 3. Results

### 3.1. Study Population Characteristics

Demographic characteristics of study population are presented in Table 1. A total of 134 eyes were recruited in this study, 100 with intermediate AMD and 34 healthy controls. Poor image quality for accurate analysis, motion and projection artefacts in OCT-A images preventing reliable evaluation were excluded. A total of 131 eyes of 104 patients were eligible for binarization and were included in the final analysis (3 eyes were excluded for insufficient image quality). Groups were divided according to drusen characteristics: 57 eyes of 52 patients presented soft or cuticular drusen (Group 1A) and 40 eyes of 28 patients presented association with SDD (Group 1B). The mean age in years in the study sample was 77.67. Groups 1 and 2 were age matched: Group 1: 78.62 ± 8.59 and Group 2: 75.72 ± 8.30. The majority of patients were women (67.2%).

### 3.2. Choroid Evaluation in Intermediate AMD

We studied OCT images with EDI mode for all recruited patients and considered choroid vascular parameters after image binarization. We found a mean TCA of 0.88 ± 0.50 mm^2^ in Group 1 and 1.24 ± 0.47 mm^2^ in Group 2. Luminal area (LA) was also reduced in Group 1 (0.40 ± 0.22 mm^2^) compared with Group 2 (0.55 ± 0.19 mm^2^). Mann-Whitney non-parametric test (Wilcoxon rank-sum test for clustered data) confirmed statistically significant differences in TCA and LA between Group 1 and Group 2 (*p* < 0.05). CVI obtained from the ratio of LA/TCA revealed lower values in Group 1 (45.3%) than in Group 2 (45.6%) with no statistical significance.

**Figure 3:**
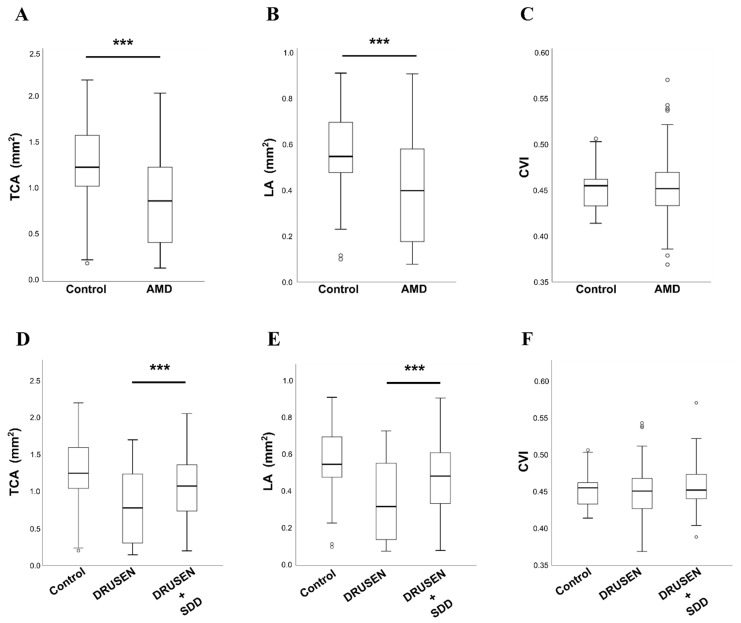
Choroid evaluation in intermediate AMD. (**A**–**C**) Graphical distribution of total choroidal area (TCA), luminal area (LA) and choroidal vascularity index (CVI), respectively, among control and AMD groups through box plots. (**D**–**F**) Box plots displayed the distribution of TCA, LA and CVI, respectively, among the control group and the two sub-groups (i.e., DRUSEN and DRUSEN + SDD) defined among AMD patients. Box-and whisker plots show median, first and third quartile, the whiskers extend to 1.5 times the interquartile range (IQR) (values exceeding this are plotted as individual points). TCA: Total choroidal area; LA: Luminal area; CVI: Choroidal vascularity index; DRUSEN: soft or cuticular drusen; DRUSEN + SDD: drusen in association with subretinal drusenoid deposits. (*** *p*-value ≤ 0.001).

Concerning subgroup analysis, the comparison between 1A versus 1B using Mann-Whitney or Wilcoxon nonparametric test showed significant differences in choroidal evaluation studied by TCA and LA with significant lower values in drusen group (Group 1A) than in those associated with SDD (Group 1B) (Figure 3 and Supplementary Table S1).

### 3.3. Retinal Capillary Plexus and Choriocapillaris Evaluation in Intermediate AMD

**Figure 4:**
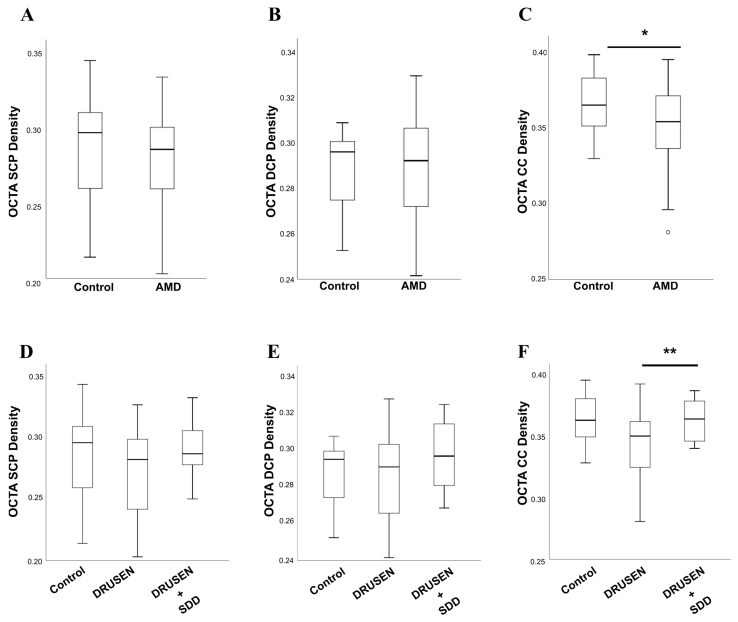
Retinal Capillary Plexus and Choriocapillaris evaluation in intermediate AMD. (**A**–**C**) Graphical distribution of SCP, DCP and CC flow density obtained by OCTA among control and AMD groups through box plots, respectively. (**D**–**F**) Box plots showed the distribution of SVP, DVP and CC flow density among the control group and the two sub-groups (i.e., DRUSEN and DRUSEN + SDD) defined among AMD patients, respectively. Box-and whisker plots show median, first and third quartile, the whiskers extend to 1.5 times the interquartile range (IQR) (values exceeding this are plotted as individual points). SCP: Superficial capillary plexus; DCP: Deep capillary plexus; CC: Choriocapillaris; FD: Flow density; DRUSEN: soft or cuticular drusen; DRUSEN + SDD: drusen in association with subretinal drusenoid deposits (* *p*-value <0.05; ** *p*-value ≤ 0.01).

Superficial and deep retinal capillary plexus presented no significant differences in FD between Group 1 and Group 2 (Figure 4 and Supplementary Table S1). We recorded reduced choriocapillary flow density in Group 1 (0.34 ± 0.02) compared with healthy controls (0.36 ± 0.02), with statistically significance in t-student test (*p* = 0.040). Group and subgroup analysis with ANOVA unidirectional test (*p* < 0.05) found statistically differences in choriocapillary flow density between groups and in subgroups (Figure 4 and Supplementary Table S1). Group 1A presented the lowest values in choriocapillary flow density.

## 4. Discussion

One main conclusion of this work is that total subfoveal choroidal area (TCA) and luminal area (LA) were reduced in intermediate AMD, compared with age matched healthy controls, supporting the concept that the choroidal vascular network is impaired in AMD patients.

**Figure 5:**
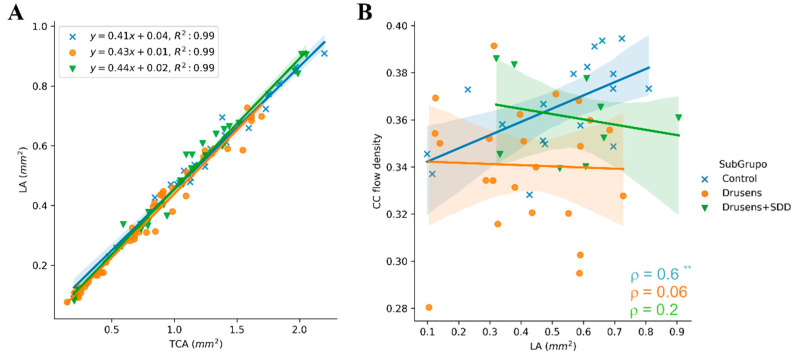
Correlation analysis with (**A**) Scatter plots with linear regression lines and coefficient of determination (R-squared) calculated for the total choroidal area and luminal area among control, 1A (Drusens) and 1B (Drusens + SDD) groups. Linear regression lines (blue-control; orange—group 1A and green-group 1B) were calculated and represented using stats’ linregress and seaborn’s lmplot functions, respectively. Light blue-, orange-, and green-coloured regions indicate the 95% confidence interval for each regression line; (**B**) Correlation scatter plots between choriocapillary (CC) flow density and luminal area (LA) (mm^2^) in control, 1A (Drusens) and 1B (Drusens + SDD) groups. Best-fitted lines (blue-control; orange—group 1A and green-group 1B) were represented and visualized through seaborn’s lmplot function. Light blue-, orange-, and green-coloured regions indicate the 95% confidence interval for each regression line. Spearman’s rank correlation coefficient (*ρ*) for each group is in the lower right corner. The correlation coefficients were: 0.6 (control), −0.06 (group 1A) and −0.2 (group 1B). The asterisks represent the *p*-value of the statistical test: ** *p*-value < 0.01.

The significant differences in TCA and LA between healthy and AMD patients corroborate the possibility of reduced choroidal perfusion and insufficient nourishing of the RPE in intermediate AMD [19]. In our cohort, CVI was also decreased in AMD patients but with no statistical significance, meaning that the decrease in luminal area was not so meaningful as the decrease in TCA. Besides, it was found that TCA and LA were linearly correlated in all groups (i.e., controls, group1A and group 1B), evidenced by the high coefficient of determination (R-squared) of 0.99 (Figure 5A). Additionally, the slopes of the regression lines were very similar among controls and intermediate AMD patients (either in group 1A or group 1B), which can explain why no statistically significant difference was found in CVI calculations for controls and AMD patients, even though a decrease in the CVI of AMD patients was perceived. Note that, CVI is the ratio between the two variables plotted, therefore the slope of the regression equation can be a rough indicator of the CVI mean for each group. Such fact could indicate that the decrease in luminal area was not so meaningful as the decrease in TCA in diseased groups.

Other authors have also studied those parameters in patients with geographic atrophy (GA) and found significant lower values of TCA, LA and CVI in those patients and CVI worsened over the follow up period [28]. In our intermediate AMD sample choroidal vascular status was not so disturbed as in this GA population and consequently CVI was not so decreased compared with age matched control group.

No significant differences in retinal circulation were found in intermediate AMD patients as compared with age-matched healthy controls. Our findings should be distinguished from other studies that found reduced vascular density in the superficial capillary plexus but a nonsignificant reduction in the deep capillary plexus in intermediate AMD patients compared with normal eyes [29].

Interestingly, choriocapillaris layer had significant lower density in AMD patients compared with controls and we found the smallest values in Group 1A; the coexistence of SDD in Group 1B was associated with improved choriocapillary density, compared with group 1A. Furthermore, the correlation between CC flow density and choroidal luminal area are very distinct in all groups. Among healthy subjects, a strong positive monotonic correlation was observed (*ρ* = 0.60, *p* < 0.01), whereas in Group 1A and 1B, the correlation between these parameters was negligible or weak and not statistically significant (Group 1A—*ρ* = −0.06 and Group 1B—*ρ* = −0.2, *p* > 0.05) (Figure 5B). The CC flow density accompanies the increase in the choroidal luminal area for control subjects, which it is not observed in intermediate AMD groups, i.e., an increase in luminal choroidal area is not followed by increase in the CC flow density in AMD patients. Considering such, one may hypothesize that AMD affects in a more significant manner the choriocapillaris vascularization than the entire choroid. This consideration and graphic relationship between CC flow density and choroidal luminal area is an interesting tool and its representativeness may constitute a challenging novel concept.

We hypothesize that drusen may act as a hydrophobic barrier, impeding the passage of fluid and nutrients between the choroid and the outer retina, resulting in relative ischemia [19]. Once the disease begins, anatomic transformation of the choriocapillaris and retinal capillary plexus, causes further hemodynamic alterations, which in turn exacerbate disease progression. These vascular features can be considered as coadjuvant factors for AMD progression or eventually trigger factors, being the choriocapillaris dysfunction the first step for the subsequent pathogenic process.

In the healthy retina, oxygen diffusion from the choroidal vasculature to photoreceptors is supposed to be perpendicular to the retinal axes. BrM thickening and drusen deposition in macular area, are changes that induce oxidative stress in such a region, consistently exposed to light [20,21]. Some models have proposed that drusen can decrease oxygen levels directly above, however allowing lateral diffusion down the concentration gradient [21,30]. This lateral transport explains why tall drusen can fail to induce severe hypoxia if they are relatively narrow to enable lateral diffusion; on the contrary thinner drusen can induce more complete hypoxia if they are large enough in lateral dimensions [31]. This appears to be the case for SDD whose coalescence in a interlacing network might prevent lateral oxygen diffusion and worsening final hypoxia, and this could be one reason for their worse prognosis [11,12]. Oxygen diffusion from outer to inner layers or on lateral direction can, as a compensation mechanism, modify the final impact of ischemia and influence our findings in vasculature impairment. Choroidal vascular and stromal characterization studies in eyes with SDD have showed some confusing results that need further clarification. Velaga et al. found thinner choroid and higher CVI in eyes with SDD but the real meaning of those parameters in final choroidal vascularity are not clear [24]. In this study, patients with SDD presented significantly higher CVI values compared to those with nonneovascular AMD patients without SDD, and healthy patients presented the lowest values. These results are quite different from those obtained in our study although the study populations do not have completely overlapping characteristics.

Functional vascular studies were already the subject of previous reports. Laser Doppler flowmetry and interferometry have been used to study foveolar choroidal blood volume, velocity, and flow [19], in the fovea. Flow in the non-exudative AMD is, on average, lower than in age-matched controls. Similar methodology showed a marked disturbance of macular retinal capillary blood flow in patients with advanced AMD [32], indicating a reduced macular perfusion in the patients with the disciform end stage of late AMD. Previous published studies have documented a decreased vascular perfusion in drusen surrounding areas [31] and demonstrated, in smaller samples, that both superficial and deep retinal plexus are changed among patients affected by the disease. These findings bring further evidence that pathological AMD-induced changes may not be limited to the outer retina but involve the inner retina as well. Nevertheless, possible association between macular perfusion and AMD does not clarify any potential cause-effect relationship.

The present study demonstrated certain limitations. Although the sole focus of the analysis were structural parameters, a further structure-function assessment would potentially provide additional valuable insights into our understanding of vascular role in AMD pathophysiology. In vivo imaging of the choriocapillaris can be achieved with OCT-A, but the presence of artifacts in the outer layers continues to be a relevant limiting factor. Furthermore, diurnal variation and hydration status may have impact in the reliability of the obtained results. Finally, the inherent subjectivity associated with manual measurements and the downsides of image binarization techniques, such as under or overestimation of hypo and hyper reflective areas, must also be considered as limiting factors [33]. The possibility of some bias in the inclusion of two eyes of the same patient, in AMD and healthy control group, can also be considered a potential weakness. Largest subgroup samples could eventually allow more solid and significant conclusions.

Studying AMD vascular pathophysiology is a relevant issue because future therapeutic approaches may target this vascular dysfunction and prevent or delay disease progression in intermediate AMD stages. Additionally, since morphological scores for predictive AMD prognosis are under investigation [34], retinal and choroidal vascular parameters could be attractive new parameters to evaluate. Choroidal vascularity index, as a measure of choroidal vascular status, has been proposed as surrogate marker for monitoring atrophic progression in AMD patients [28].

In the present study total subfoveal choroidal area, luminal area and choriocapillary flow density, seem to reveal themselves as attractive vascular parameters in intermediate AMD. Further studies are needed to evaluate their role as prognostic biomarkers, to propose other more and to compare them with existing prognostic factors.

## Figures and Tables

**Table 1:** Demographic characteristics of study population.

	Total	Amd Group 1	DRUSEN Group 1A	DRUSEN + SDD Group 1B	Control Group 2
*n*	131	97	57	40	34
%	100%	74.0%	43.5%	30.5%	26.0%
GENDER (M/F)	(43/88)	(39/58)	(22/37)	(18/22)	(5/29)
AGE	77.67 ± 8.59	78.62 ± 8.59	78.58 ± 7.38	78.67 ± 8.25	75.72 ± 8.30

M: Male; F: Female; DRUSEN: soft or cuticular drusen; DRUSEN + SDD: drusen in association with subretinal drusenoid deposits.

## Data Availability

All relevant data are within the paper and its Supporting Information file.

## Supplementary Materials

The following supporting information can be downloaded at: https://www.mdpi.com/article/10.3390/diagnostics12051290/s1, Table S1: Study Data Base.
