# Supplementary material for: Choroidal Vascular Impairment in Intermediate Age-Related Macular Degeneration
Source: Diagnostics (Basel). 2022 May 22;12(5):1290. doi: 10.3390/diagnostics12051290 (PMC9141612; doi:10.3390/diagnostics12051290)
Supplement: Supplementary file 1 [file diagnostics-12-01290-s001.zip › diagnostics-1719137-supplementary.pdf]

Supplementary Data

NUM	Gender	GROUPs	sub GROUPs	EYE	AGE	TCAmm	CLAmm	CVI	OCTA SVP Density	OCTA DVP Density	OCTA CC Density
1	2	1	1	OE	76	1.0764	0.5172	0.4805			
2	1	1	1	OE	66	0.9715	0.4698	0.4836	0.2583	0.2734	0.3512
3	1	1	1	OE	88	1.0210	0.4715	0.4617	0.2683	0.2880	0.3667
4	1	1	1	OE	74	1.7308	0.7231	0.4178	0.3038	0.3023	0.3946
5	1	1	1	OD	85				0.2137	0.2586	0.3393
6	2	1	1	OD	82	1.5936	0.6596	0.4139	0.2944	0.3003	0.3936
7	1	1	1	OE	75	1.2871	0.5900	0.4584	0.3094	0.2958	0.3577
8	2	1	1	OD	82	1.2486	0.5681	0.4550			
9	2	1	1	OD	77	0.4753	0.2290	0.4817	0.3050	0.2938	0.3729
10	1	1	1	OE	77	1.7529	0.7733	0.4412			
12	1	1	1	OE	79	0.1973	0.0985	0.4993	0.2852	0.2856	0.3455
13	1	1	1	OD	76	0.8441	0.4270	0.5059	0.2448	0.2618	0.3282
14	1	1	1	OD	70	1.2486	0.5681	0.4550	0.3411	0.3061	0.3796
15	1	1	1	OD	45	1.1093	0.5098	0.4596			
16	1	1	1	OD	83	1.0382	0.4761	0.4586	0.2819	0.2808	0.3497
17	1	1	1	OE	63	1.9686	0.8637	0.4388			
18	1	1	1	OD	71	1.3824	0.6951	0.5028	0.3074	0.2951	0.3732
19	1	1	1	OD	79	1.1612	0.5362	0.4617			
21	1	1	1	OE	81	0.7466	0.3395	0.4547	0.2812	0.2756	0.3581
22	1	1	1	OE	69	1.2357	0.5461	0.4419			
23	1	1	1	OE	84	1.4140	0.6120	0.4328	0.3161	0.3049	0.3825
24	1	1	1	OD	80	1.1316	0.4922	0.4349			
25	2	1	1	OE	82	1.6162	0.6955	0.4303	0.2257	0.2718	0.3487
27	1	1	1	OD	79	0.2346	0.1151	0.4906	0.2555	0.2579	0.3371
28	1	1	1	OE	76	1.1245	0.4791	0.4261			
29	1	1	1	OE	70	1.6162	0.6955	0.4303	0.3411	0.3061	0.3796
30	1	1	1	OE	83	1.2453	0.5301	0.4257			
31	1	1	1	OD	63	2.1973	0.9093	0.4138			
32	1	1	1	OE	71	1.8372	0.8090	0.4404	0.3074	0.2951	0.3732
33	1	1	1	OD	81				0.3098	0.2981	0.3622
34	1	1	1	OD	69	1.4027	0.6353	0.4529	0.2993	0.2976	0.3912
35	1	2	2	OE	81	0.3833	0.1660	0.4331			
36	1	2	2	OD	83	0.6456	0.2821	0.4370			
37	1	2	2	OE	84	0.6834	0.2978	0.4358	0.2844	0.2707	0.3521
38	2	2	2	OE	81	0.2842	0.1280	0.4503			
39	2	2	2	OE	84	0.2286	0.1240	0.5425	0.2964	0.3259	0.3542
40	2	2	2	OE	83	0.9113	0.4475	0.4911	0.2421	0.2665	0.3399
41	1	2	2	OE	64	1.6154	0.6837	0.4233	0.2702	0.2903	0.3557
42	1	2	2	OD	78	0.8409	0.4081	0.4853	0.2978	0.2899	0.3510
43	2	2	2	OE	77	0.2076	0.1043	0.5026	0.2073	0.2414	0.2804
44	1	2	2	OE	78	1.4043	0.6213	0.4424	0.3066	0.3007	0.3598
45	2	2	2	OE	77	0.4171	0.1752	0.4202			
46	2	2	2	OE	82	0.9070	0.4326	0.4769			
47	2	2	2	OE	79	0.2273	0.0967	0.4255			
48	2	2	2	OD	85	1.6162	0.6955	0.4303			
49	2	2	2	OE	90	1.2325	0.5630	0.4568			
51	1	2	2	OD	93	0.9834	0.4596	0.4674			
52	1	2	2	OD	85	1.0928	0.4322	0.3955			
53	1	2	2	OE	68				0.2914	0.3038	0.3805
54	2	2	2	OD	68	0.8482	0.3128	0.3688	0.3220	0.3171	0.3915
55	2	2	2	OE	79	0.6912	0.3173	0.4590			
56	1	2	2	OD	88	0.9850	0.3799	0.3857	0.2206	0.2568	0.3314
57	1	2	2	OE	87	0.2255	0.1028	0.4557			
58	1	2	2	OD	75	1.6966	0.6981	0.4115			
59	1	2	2	OD	85	0.6627	0.3251	0.4905	0.2372	0.2593	0.3158
60	1	2	2	OE	73	1.3223	0.5871	0.4440	0.3129	0.2953	0.2949
61	1	2	2	OE	70	1.4791	0.6545	0.4425			
62	1	2	2	OE	88	0.1433	0.0769	0.5366			
63	1	2	2	OE	83	0.2132	0.0970	0.4552			
64	1	2	2	OD	90	1.2330	0.5517	0.4474	0.2686	0.2834	0.3203
65	1	2	2	OD	75	1.3131	0.5896	0.4490	0.2030	0.2474	0.3027
66	1	2	2	OD	75	1.5462	0.5855	0.3787	0.2828	0.2930	0.3681
67	1	2	2	OD	75	0.7184	0.3105	0.4322			
69	1	2	2	OE	75	1.2433	0.5840	0.4697			
70	2	2	2	OE	73	0.9003	0.4395	0.4882			

Supplementary Data

71	1	2	2	OD	85	1.4253	0.5900	0.4140	0.2401	0.2637	0.3488
72	1	2	2	OD	81	0.8662	0.3978	0.4593			
73	1	2	2	OD	77	0.8290	0.3964	0.4782	0.2580	0.2861	0.3624
74	1	2	2	OE	78	1.5826	0.7272	0.4595	0.2141	0.2521	0.3278
76	2	2	2	OD	68	1.0999	0.5114	0.4649	0.3247	0.3041	0.3710
77	2	2	2	OE	59	0.6790	0.2873	0.4231	0.2856	0.3027	0.3343
78	1	2	2	OD	66	0.8872	0.4350	0.4903	0.2790	0.2835	0.3206
79	1	2	2	OD	81	0.4526	0.2094	0.4626			
80	2	2	2	OD	81	0.3148	0.1454	0.4618			
81	2	2	2	OD	84	0.2454	0.1255	0.5112	0.2996	0.3040	0.3693
82	2	2	2	OD	77	0.3013	0.1392	0.4621	0.2907	0.2946	0.3501
83	2	2	2	OD	77	0.4325	0.1756	0.4060			
84	2	2	2	OD	77	0.2521	0.1076	0.4268			
85	1	2	2	OE	82	1.2993	0.5744	0.4421			
86	1	2	2	OD	87	0.2337	0.0917	0.3923			
87	1	2	2	OE	92	0.2309	0.1015	0.4394			
88	1	2	2	OD	88	0.2009	0.1082	0.5385			
89	1	2	2	OD	83	0.3698	0.1675	0.4530			
90	1	2	2	OE	83	0.2556	0.1191	0.4660			
91	2	2	2	OD	59	0.7766	0.3099	0.3990	0.2609	0.2896	0.3343
92	2	2	3	OD	80	2.0528	0.9052	0.4410	0.2612	0.2799	0.3609
93	1	2	3	OE	85	0.5383	0.2576	0.4786			
94	2	2	3	OD	78	0.5835	0.2634	0.4514			
95	2	2	3	OE	81	0.7343	0.3205	0.4365	0.2868	0.2999	0.3860
96	2	2	3	OE	89	1.3334	0.6392	0.4794			
97	2	2	3	OE	82	0.9146	0.4029	0.4405			
98	1	2	3	OE	74	2.0164	0.9060	0.4493			
99	2	2	3	OD	82	0.5911	0.3368	0.5699			
100	2	2	3	OE	54	1.0713	0.4713	0.4399			
101	1	2	3	OD	81	1.1364	0.5237	0.4608	0.2493	0.2679	0.3394
102	1	2	3	OD	88	0.1958	0.0885	0.4518			
103	1	2	3	OE	79	0.9410	0.3653	0.3882			
104	1	2	3	OD	93	0.5423	0.2512	0.4632			
105	2	2	3	OE	76	1.9862	0.8416	0.4237			
106	2	2	3	OE	65	1.4384	0.6651	0.4624	0.2971	0.3090	0.3524
107	2	2	3	OE	77	1.4026	0.6101	0.4350	0.2767	0.2792	0.3777
108	2	2	3	OE	77	1.0477	0.4828	0.4608			
109	2	2	3	OD	72	1.1420	0.5698	0.4990			
110	2	2	3	OD	76	1.0577	0.4815	0.4552			
111	1	2	3	OD	78	1.3981	0.6552	0.4686	0.3304	0.3230	0.3654
113	1	2	3	OE	93	1.0943	0.5704	0.5213			
115	2	2	3	OE	79	1.2486	0.5681	0.4550			
116	2	2	3	OE	83	0.7315	0.3362	0.4596			
117	1	2	3	OE	73	0.7926	0.3779	0.4768	0.3040	0.3126	0.3835
118	1	2	3	OD	86	0.7888	0.3319	0.4208	0.2842	0.2911	0.3454
119	1	2	3	OE	87	0.2122	0.1021	0.4809			
120	1	2	3	OD	75	0.7961	0.3503	0.4400			
121	1	2	3	OD	83				0.2795	0.2821	0.3742
122	2	2	3	OD	54	1.3842	0.5987	0.4326			
123	1	2	3	OE	88	0.1993	0.0805	0.4039			
124	2	2	3	OD	65	1.5157	0.6762	0.4462			
125	1	2	3	OE	78	1.9635	0.8505	0.4332			
127	1	2	3	OD	93	1.2256	0.6075	0.4957	0.3155	0.3164	0.3401
128	1	2	3	OE	86	1.1518	0.5095	0.4424			
129	2	2	3	OD	83	1.1834	0.5302	0.4481			
130	1	2	3	OD	87	0.2313	0.1175	0.5081			
